# Application of lung microphysiological systems to COVID-19 modeling and drug discovery: a review

**DOI:** 10.1007/s42242-021-00136-5

**Published:** 2021-06-18

**Authors:** Argus M. Sun, Tyler Hoffman, Bao Q. Luu, Nureddin Ashammakhi, Song Li

**Affiliations:** 1 Department of Bioengineering, Samueli School of Engineering, University of California - Los Angeles, 420 Westwood Plaza 5121 Engineering V University of California, Los Angeles, CA 90095-1600 USA; 2 UC San Diego Healthcare, UCSD, La Jolla, CA 92037 USA; 3 Pulmonary Diseases and Critical Care, Scripps Green Hospital, Scripps Health, La Jolla, CA 92037 USA; 4 Department of Biomedical Engineering, College of Engineering, Michigan State University, East Lansing, MI 48824 USA; 5 Department of Medicine, David Geffen School of Medicine, UCLA, Los Angeles, CA 90095 USA

**Keywords:** Bioengineering, Microfluidics, COVID-19, Organ-on-a-chip, Lung, Microvascular networks, Drug development

## Abstract

There is a pressing need for effective therapeutics for coronavirus disease 2019 (COVID-19), the respiratory disease caused by severe acute respiratory syndrome coronavirus 2 (SARS-CoV-2) virus. The process of drug development is a costly and meticulously paced process, where progress is often hindered by the failure of initially promising leads. To aid this challenge, in vitro human microphysiological systems need to be refined and adapted for mechanistic studies and drug screening, thereby saving valuable time and resources during a pandemic crisis. The SARS-CoV-2 virus attacks the lung, an organ where the unique three-dimensional (3D) structure of its functional units is critical for proper respiratory function. The in vitro lung models essentially recapitulate the distinct tissue structure and the dynamic mechanical and biological interactions between different cell types. Current model systems include Transwell, organoid and organ-on-a-chip or microphysiological systems (MPSs). We review models that have direct relevance toward modeling the pathology of COVID-19, including the processes of inflammation, edema, coagulation, as well as lung immune function. We also consider the practical issues that may influence the design and fabrication of MPS. The role of lung MPS is addressed in the context of multi-organ models, and it is discussed how high-throughput screening and artificial intelligence can be integrated with lung MPS to accelerate drug development for COVID-19 and other infectious diseases.

## Introduction

At the time of this review, coronavirus disease 2019 (COVID-19), the disease caused by severe acute respiratory syndrome coronavirus 2 (SARS-CoV-2) virus, has infected over 54 million people globally following its emergence from Wuhan, China, in December 2019 [1]. Complications related to COVID-19 have resulted in over a million deaths, serious morbidity in many surviving patients, and economic havoc throughout the world [2–8]. Although collaborative and multidisciplinary research efforts have sped up the development and repositioning of drugs against COVID-19 [9–11], no effective therapy has been established to date. Given the urgent need and limited resources, screening lead compounds with microphysiological systems (MPSs) or organ-on-a-chip systems can offer many advantages over traditional methods for testing the safety and efficacy of novel or repositioned drugs and for investigating the mechanisms of action of said drugs. Pharmaceutics for COVID-19 can be categorized into therapeutics for the inhibition of viral entry (by targeting SARS-CoV-2 spike proteins and associated receptors and mediators), blockage of virus replication, treatment of infection-related inflammation (maintenance of tolerable cytokine levels), prevention of coagulation and thrombosis in infected tissues, prevention or reversal of alveolar flooding, and prevention of fibrosis. The in vitro recapitulation of drug-targeted pathological processes requires the in-depth understanding of disease states at the cellular and tissue level. For instance, mortality in COVID-19 is often due to alveolar flooding, which has three main causes: failure of epithelial barrier, inhibition of amiloride-sensitive Na^+^ channels in the alveolar epithelium (thereby disrupting absorption of liquid from the alveolar lumen), or loss of surfactant function. Through modeling specific aspects of the disease state, MPS can play a key role in accelerating, de-risking, or supplementing the development of novel treatments for various pathologies. Excellent reviews have been published on general organ-on-a-chip and lung-on-a-chip models for drug development [12–14]. Herein, we provide an update on in vitro lung MPS and discuss the further development of MPS for mechanistic studies and drug screening in relevance to COVID-19.

## Modeling lung function

**Fig. 1:**
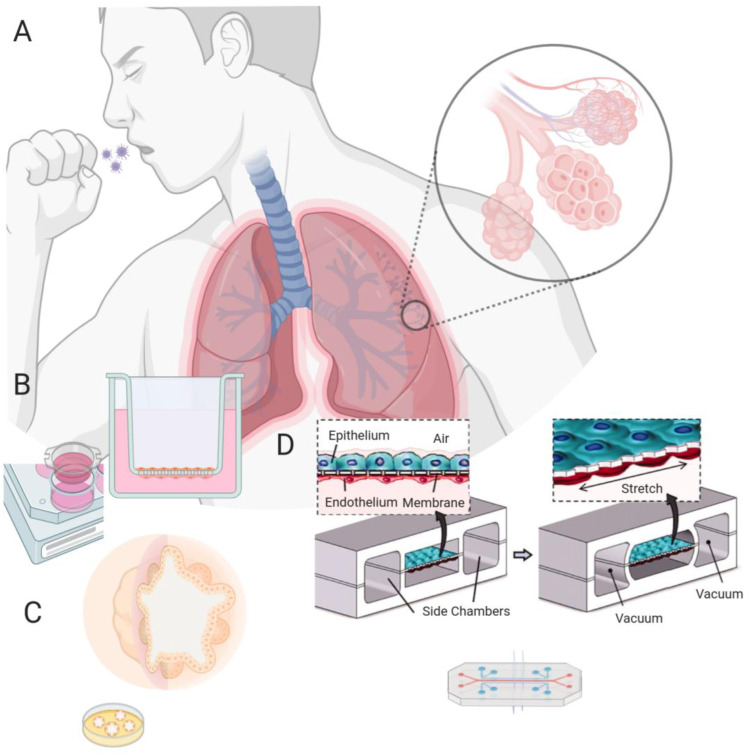
Lung microphysiological systems for COVID-19. **a** The lung has structurally unique tissue organization, characterized by a successively finer branching system of airways that terminate in the alveoli where oxygenation of blood from the circulatory system occurs. An accurate and rapidly deployable alveolar-level lung model is needed to develop drugs against the pathological damage caused by COVID-19, the disease resulting from SARS-CoV-2 viral infection. **b** Preceding the development of true lung MPS, Transwell systems used a membrane fitted insert into multiwell cell culture assay plates. Inserts with membranes that prevent fluid passage create an air–liquid interface where alveolar epithelial cells and vascular endothelial cells can be grown on each side. **c** Organoids can be formed by seeding an extracellular matrix with stem cells and then using a sequence of growth factors to differentiate them into a set of lung cells that organize spatially complex tissues resembling in vivo tissue architecture. **d** Lung microphysiological systems or lung-on-a-chip; a PDMS microdevice with a thin PDMS layer coated with ECM acting as an alveolar epithelial–capillary border. Breathing motions are recreated by applying vacuum to side compartments generating mechanical stretch of the alveolar–capillary membrane. Reproduced with permission from Huh et al. [19], Copyright 2010

In the past two decades, the in vitro modeling of cellular functions and interactions in lung tissues has evolved from a relatively simple co-culture setting to MPS that mimics the biomechanical, biochemical, and biological factors in the lung, as exemplified by Transwell systems, organoids, and MPS (Fig. 1).

### Transwell systems

Alveolar epithelial cells can be cultivated in a two-dimensional (2D) culture on extracellular matrix (ECM) coated or uncoated tissue culture plastics. Such 2D culture systems can be modified with the placement of a Transwell insert, elevating the culture onto a semipermeable membrane above the floor of the well and thus creating a media reservoir beneath the membrane. The new surfaces created in the system, such as the now vacant well floor and the underside of the insert membrane, can be used to culture additional cell types, such as endothelial cells (Fig. 1b). Transwell systems also allow the epithelial cell layer to grow on an air–liquid interface, allowing epithelial differentiation toward mucous-secreting goblet cells [15]. A proof-of-concept study using dextrans and fluorescent dyes showed that although the permeability of the alveolar cell-occupied membrane was lower in Transwell systems compared to later discussed microfluidic MPS devices, the barrier permeability to 70-kDa dextran was similar in both systems [16]. In a similar manner, to determine the prevailing type of transepithelial transport, the transmural voltage (*V*_te_) can be measured with voltage-sensing fluorescent probes. Small fluorescent probes could also be used to determine the integrity of tight junctions. Transepithelial electrical resistance (TEER), an index of barrier function and amiloride-sensitive *V*_te_ (an index of the epithelium’s ability to remove fluid from the alveolar lumen), can be readily measured by a voltmeter equipped with specialized fixed width double electrodes. The basolateral and apical media can be sampled for released compounds. With regard to ultrastructure, cells grown on inserts may show similar differentiation to those grown in other systems with an air–liquid interface [17]. Transwell systems are advantageous because their relative simplicity allows for rapid deployment in sets of 48 inserts. An issue with Transwell culture is that the lack of microfluidic flow does not induce physiological shear stress or pressure on cells, which can result in a difference in permeability between compartments compared to fluidic-stressed cells.

### Organoids

The widespread use of organoids began in 2009, when Hans Clevers and colleagues grew a self-organizing structure from stem cells that differentiated into tissues with organ-like features [18]. Organoids are functional units generated by seeding pluripotent or induced pluripotent stem cells (PSC/iPSC) onto suspended clusters of collagen or ECM solution such as Matrigel (an ECM isolated from Engelbreth-Holm-Swarm (EHS) mouse sarcoma cells), and adding the appropriate growth factors for cells to differentiate into the lineage of interest in the growing organoid [19, 20]. Hollow spheres of epithelial cells form within the gel, with their apical membrane facing inwards (Fig. 1c). The spheres can be studied in situ or released by enzymatic treatment to form a suspension. The types and magnitudes of transepithelial ion transport processes present can be determined based on changes in the volume of individual organoids. For instance, cyclic adenosine monophosphate (cAMP)-elevating agents cause organoids of nasal epithelium to swell, except if they are derived from patients with cystic fibrosis [21]. Therefore, the swelling is presumably due to the activation of cystic fibrosis transmembrane conductance regulator (CFTR)-dependent chloride ion (Cl^–^) secretion. Likewise, swelling upon amiloride application would indicate the presence of active sodium ion (Na^+^) absorption, the process that combats alveolar flooding. Unfortunately, the amiloride-sensitive Na^+^ channels are in the apical membrane, while the 229-Da amiloride molecule is small enough for sufficient amounts of it to diffuse across the tight junctions. Based on the differentiation characteristics of tissue-engineered lung, organoids are subdivided into tracheospheres, bronchospheres, or alveolospheres. Alveolospheres with both type I and type II alveolar cells can be maintained through the application of interleukin-6 (IL-6)/signal transducer and activator of transcription proteins 3 (STAT3), bone morphogenetic protein (BMP), and fibroblast growth factor (FGF) [19]. Clustered regularly interspersed short palindromic regions (CRISPR)/CRISPR-associated protein 9 (Cas9) methods have identified a ciliation transcription factor in airway cells that may be useful for the knock-in/knock-out/knock-down identification of growth factor pathways important for cell differentiation and disease formation in organoids [22–24]. Organoids can be cultured in a variety of microenvironments with a wide range of conditions; coating lung organoids with poly(lactide-*co*-glycolide) (PLGA) allows for their implantation and maturation in vivo [25]. The drawbacks of organoids include the lack of an air-filled lumen necessary for testing inhaled therapeutics, the lack of an accessible endothelial-lined compartment simulating the vasculature essential for the pharmacokinetic study of the lung-circulation interface, as well as variability in growth, differentiation, and maturation [19, 22, 26]. A further problem with organoids is that the receptor for coronaviruses, angiotensin-converting enzyme 2 (ACE2, discussed in Sect. “ACE2 and viral entry”), is in the apical membrane. Thus, gentle enzymatic and physical disruption is needed to bring virus and receptor together [27]. Therefore, differences in the disruption procedure between studies could lead to variability in the results.

### Lung microphysiological systems

**Fig. 2:**
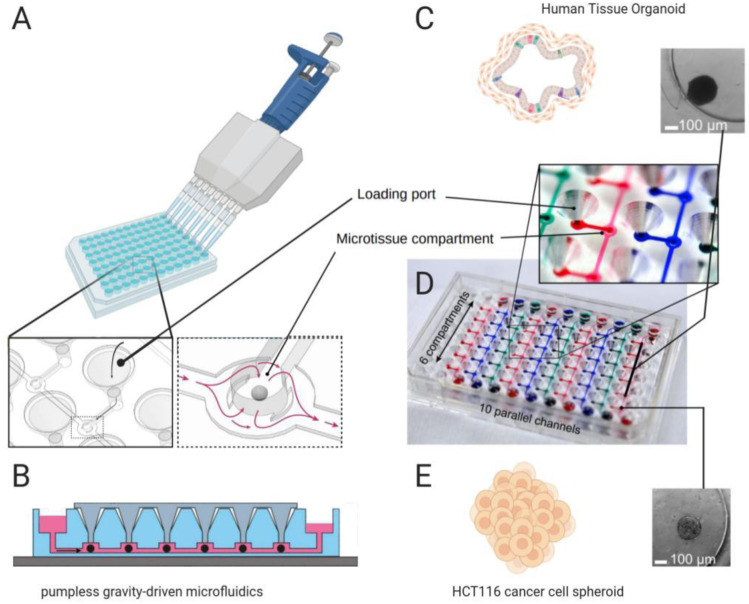
Pumpless organoid MPS.** a** Organoids can be loaded into the MPS, which is then perfused by microfluidic flow. Reproduced with permission from Frey et al. [35], Copyright 2015. **b** Pumpless flow is driven by gravity, where alternate setup allows continuous flow. **c** Human tissue organoid (hLiMT) perfused in the MPS shown at 5 × magnification. **d** Colored dye is used to show stacking of up to 60 experiments. **e** Cancer cell (HCT116) spheroid shown at 1 × magnification

Organ-on-a-chip methods (a term used interchangeably with microphysiological systems) use microfluidic flow to culture cells in an organotypic configuration. These microfluidic devices are made by microfabrication methods borrowed from the semiconductor industry, hence their name [28]. Huh et al. fabricated an organ-on-a-chip model of the lung alveolus in 2010 by etching two adjacent channels in polydimethylsiloxane (PDMS), which were separated by a 10 μm porous and flexible PDMS membrane [29]. After coating each side of the membrane with extracellular matrix, solutions containing either lung epithelial cells or vascular endothelial cells were flowed into the channels, allowing cells to colonize and expand on both sides of the membrane (Fig. 1d). Once the cells reached confluence, the growth medium from the top channel was removed to create an air–liquid interface. The flexibility of PDMS allows for cyclic stretching with air pressure changes, modeling the stress–strain pattern that occurs during in vivo breathing [29, 30]. Such stretching influences permeability and the release of reactive oxygen species, cytokines, and surfactant [29–31]. An advantage of the lung-on-a-chip systems is that other cell types can be grown separated from but in close proximity to epithelial cells, thereby mimicking in vivo interactions. Furthermore, TEER [29] and amiloride-sensitive *V*_te_ can be measured in such systems. A comparison of alveolar cells cultured under microfluidic flow without oscillatory pressure changes to simulate breathing showed higher TEER than that in a Transwell culture [32]. Stucki et al. used electrodes to simultaneously monitor TEER and epithelial movement, which enabled the modeling of a dynamic three-dimensional (3D) alveolar microenvironment [33]. A criticism of lung MPS is that the design of an individual chip is complex and may harbor the difficulty of assembly and usage in sufficient numbers for drug screening amidst a rapidly expanding threat like COVID-19. Efforts have been made to remedy this situation in the form of MPS-based startups and spinoffs [34], with a notable example shown in Fig. 2, which is an *Akura* platform by InSphero AG using pumpless microfluidic circulation to perform on-chip perfusion of organoids [35].

## Modeling lung pathology

**Fig. 3:**
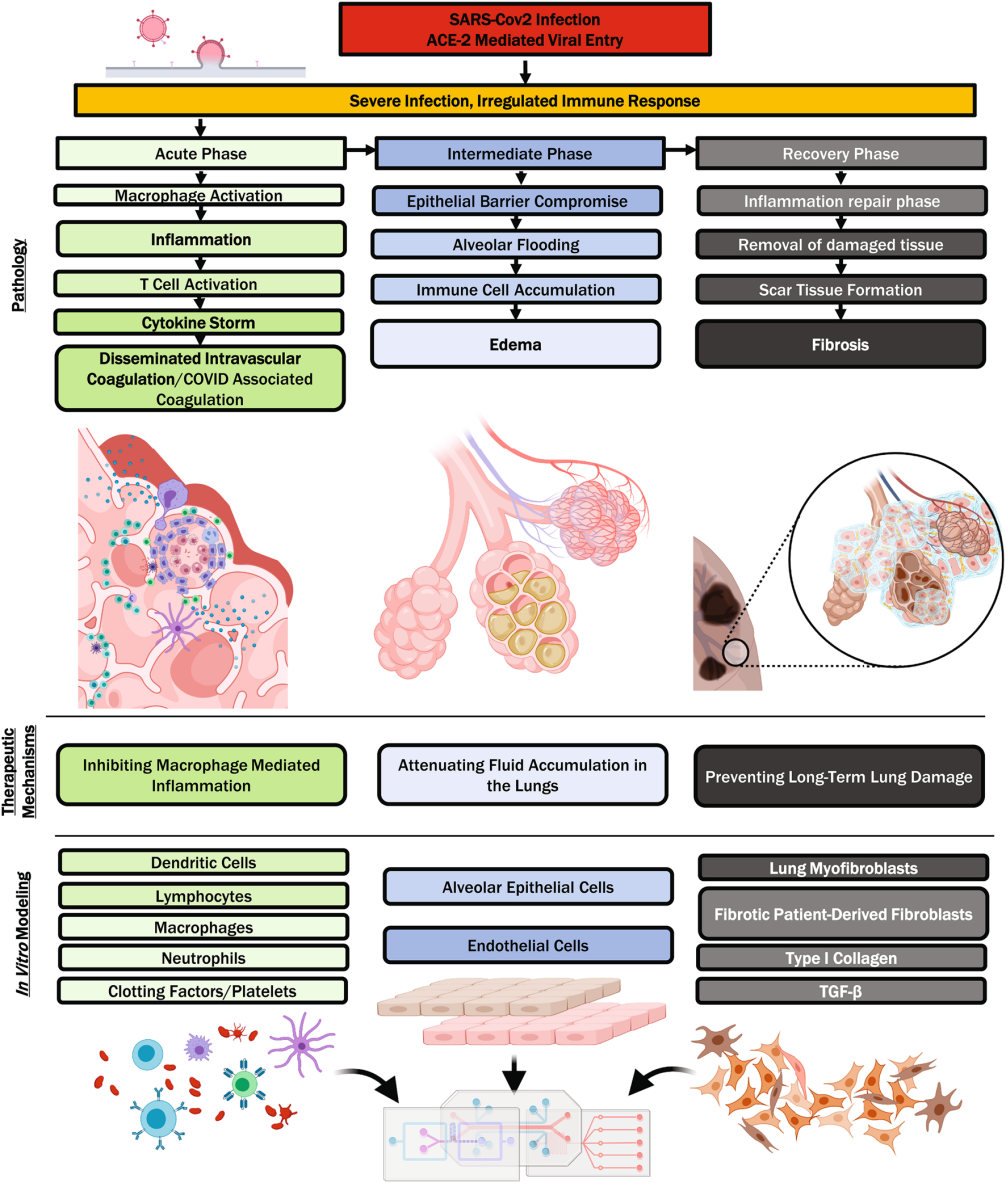
Overview of COVID pathology and therapeutics in relation to in vitro modeling. COVID-19 is generally agreed to have three phases: an acute phase characterized by inflammation, coagulopathy, and immune malfunction, an intermediate phase characterized by edema and a recovery phase characterized by the buildup of fibrotic extracellular matrix. Drug discovery for each stage presents unique challenges that can be overcome by adapting the usage of lung MPS

Several in vitro lung models exist for COVID-19 drug discovery, each with their unique advantages; however, lung MPSs are versatile and have already demonstrated usefulness in several pathological manifestations of COVID-19. In the following sections, we discuss how MPSs have aided new drug candidate testing, as well as the potential ways in which they can be of further assistance. In particular, COVID pathology can be organized into the following stages: SARS-CoV-2 viral entry by the ACE2 receptor; inflammation or malfunction of the innate immune response; coagulopathy or clotting dysregulation; edema or swelling and fluid accumulation; fibrosis or scarring through the buildup of fibrotic connective tissue (Fig. 3).

**Fig. 4:**
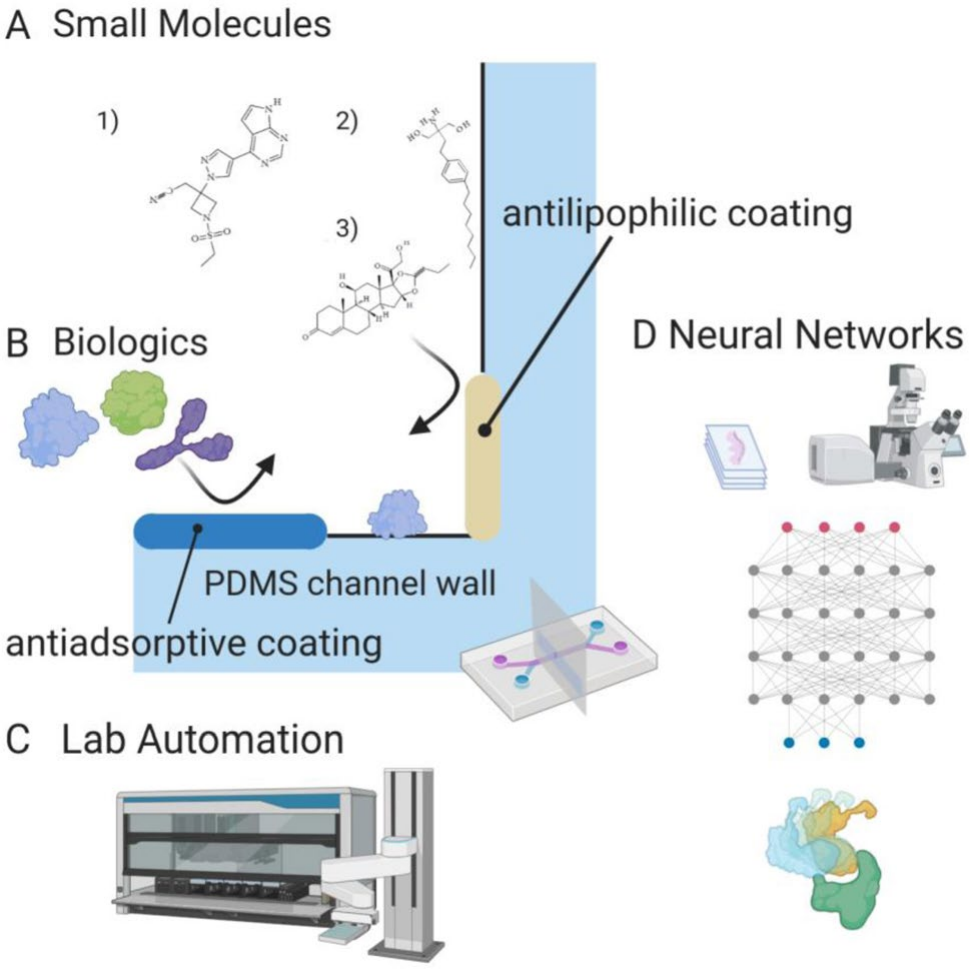
Challenges to lung MPS and upcoming advances. **a** Small, typically lipophilic molecules bind to surfaces such as PDMS channel walls and can be characterized by the Langmuir–Freundlich isotherm. Coating a PDMS surface with paralyne or using sol–gel methods can prevent lipophilic binding [156, 157]. **a1** Baricitinib, a Janus-associated kinase (JAK/STAT) inhibitor immunosuppressant, is used to treat COVID-associated hemophagocytic lymphohistiocytosis (HLH) [76]. **a2** Fingolimod, a repurposed multiple sclerosis drug [89]. Aliphatic domains such as the hydrophobic tail create opportunities for the drug to bind to channel walls. **a3** Budesonide, an anti-inflammatory steroid compared tested on lung MPS [90]. **b** Biologics such as antibodies and recombinant proteins adsorb to PDMS channel walls; methods to prevent adsorption include oxygen plasma treatment, amphilic, self-assembled monolayer and hydrophilic polymer graft coating [157, 161]. **c** Integrating MPS devices with automated liquid handling and continuous flow will introduce a new potential for streamlining drug discovery workflows and increasing throughput for screening lead compounds. **d** Machine learning and artificial intelligence algorithms such as neural networks can aid drug discovery through molecular docking and design, image analysis and toxicity predictions. Effective usage includes generating and seeking out sufficiently large datasets to train algorithms to make accurate predictions

### ACE2 and viral entry

The ACE2 enzyme is an 85.9-kDa membrane-bound zinc metalloproteinase that functions to counter-regulate the renin–angiotensin–aldosterone axis by converting angiotensin II to angiotensin I. The SARS-CoV-2 and SARS-CoV viruses gain entry to ACE2-expressing cells when the spike protein on viral particles binds to ACE2 and is endocytosed when the ACE2 is recycled. The virus then exits the endosome into the host cell cytoplasm, where it uses its RNA genome to replicate using the host cell’s protein synthesis machinery. Drugs that mechanistically target this pathway constitute a significant portion of the existing COVID treatments. Following the outbreak of SARS-CoV, 9% of papers published on therapeutics focused on ACE2. In combination with the spike protein, this total neared a third [36]. SARS-CoV-2 has 20 times greater affinity for ACE2 compared with SARS-CoV, justifying the efforts toward analyzing ACE2 targets for drug development [37]. Computational screening was shown to be effective in conjunction with cell culture to screen potential leads. In one example, a structural similarity of influenza neuraminidase to coronavirus protease 3CLPro suggested that the neuraminidase inhibitor oseltamivir has antiviral action against SARS-CoV-2. Unfortunately, molecular docking showed the inhibition to be weak, which was confirmed in a culture of ACE2-expressing Vero E6 cells [38]. Computational docking, however, has shown promising results against SARS-CoV-2 spike protein and proteases and can be useful for preliminarily narrowing down the related leads for further testing in MPSs [9, 39].

The therapeutic potential of a lead must be confirmed by showing how cells react to it and how well it performs against a cellular model of COVID-19. For a lung MPS, modeling SARS-CoV-2 infection in vitro requires the presence of lung cells expressing ACE2 and its co-receptor transmembrane protease serine 2 (TMPRSS2). The expression of both receptors is the highest in type II alveolar cells (AT2/AEC2) [40, 41]. Using a validated antibody and mRNA sequencing, ACE2 expression was found in the intestinal microvilli, renal proximal tubule, gallbladder epithelium, and also in testicular Sertoli and Leydig cells [42]. Consistent with the sequencing data, infections with SARS-CoV and other betacoronaviruses related to SARS-CoV2 were found in Vero, Caco-2, and Huh-7.5 cells, which originate from the kidney, intestine, and liver, respectively [43]. Surprisingly, ACE2 expression was not detected in the frequently studied lung epithelial cell line A549 [42]. Although there has been significant work on ACE2, controversial reports were also published on ACE2-independent viral entry using CD147/basigin [44, 45]. Primary cultures of alveolar type II cells were used in early studies of epithelial ion transport [46]. When grown in porous-bottomed inserts used in a Transwell culture, these cells form tight junctions (as reflected in high transepithelial resistance, TEER), polarize, and display amiloride-sensitive Na^+^ transport [47]. The earliest such cultures dedifferentiated rapidly with time and ultrastructurally changed to resemble type I alveolar cells [48]. Several approaches to preventing this dedifferentiation were described later [49–53], with perhaps the most effective one, in terms of ultrastructure and surfactant production, being the use of an air interface [54]. This approach, however, has little effect on amiloride-sensitive Na^+^ transport [55], which is consistent with studies on isolated type I cells in that they also had the necessary apparatus for this transport process [56, 57]. No culture has yet been derived from type I cells, while a cell line that resembles them, hAELVi, has been generated from human alveolar epithelium [58]. Cells relevant to the compound being evaluated should be included when designing a model, although caution should be taken against the inclusion of too many extraneous cell types that might unnecessarily complicate the model.

The MPS-related methods have also been employed in COVID infection models with non-lung cells. One such example is the testing of human recombinant soluble ACE2 (hrsACE2) as a potential biologic treatment against SARS-CoV-2 infection, which works by binding to the viral spike protein blocking its interaction with cellular ACE2. Infection was reduced by several orders of magnitude in Vero cell culture and kidney organoids (Table 1) [59]. Using a similar strategy with SARS-CoV-2-neutralizing monoclonal antibodies, lung pathology was prevented in hamsters [60]. Targeting the TMPRSS2 co-receptor with the serine protease inhibitor camostat mesylate is thought to be a further potential means to prevent viral entry into cells [61]. Interestingly, inflammation due to a depletion of ACE2-expressing cells is partially ameliorated by the estrogen-mediated upregulation of ACE2, possibly accounting for sex differences in mortality and making estrogen a component of COVID therapeutic regime [62]. The unique modifications needed for MPSs when used for steroid-like small molecules (summarized in Table 1) are discussed in “Current challenges and future perspectives”.

**Table 1:** COVID therapeutic categories for MPS screening

Therapeutic	Type	Mechanism	Examples	MPS notes	References
Recombinant; Receptor	Biologic	Viral binding	hrsACE2	Antiadsorp; Channel coating	[59, 60]
JAK/STAT inhibitors	Small molecule	IL-6 pathway	Baricitinib; Ruxolitinib	Antilipophilic; Channel coating	[80, 81, 83, 89, 90]
Corticosteroid	Small molecule	Inflammation; edema	Dexamethasone; Budesonide	Antilipophilic; channel coating	[76, 94]
Nanomedicine	Nanoparticle	Varies with cargo	PEG-liposomal; Dexamethasone	Varies	[94, 134, 156]
TRPV4 inhibitor	Small molecule	Edema	GSK2798745	Antilipophilic; channel coating	[110, 156, 157]
S1PR antagonist	Small molecule	Inflammation	Fingolimod	Antilipophilic; Channel coating	[78, 89]
Heparin	Polysaccharide	Anticoagulant	UFH; enoxaparin; Dalteparin	Adj flow for viscosity; Shear	[93, 95, 96]
PAR-1 inhibitor	Small molecule	Anticoagulant	PM-2	Antilipophilic channel coating	[77, 102]
Antifibrotic	Small molecule	Antifibrotic; inflammation	Pirfenidone; nintedanib	Antilipophilic; channel coating	[111, 116]
Cytokine inhibitor	Antibody	Inflammation	Tocilizumab; Sarilumab; Ustekinumab	Antiadsorp; Channel coating	[80, 82, 84, 85, 89]
TLR-4 inhibitor	Small molecule	Inflammation	Eritoran	Antilipophilic; channel coating; creatinine flow media	[86, 87, 141, 142]
C5 inhibitor	Antibody	Anticoagulant	Eculizumab	Antiadsorp; Channel coating	[97, 157, 161–164]
PDE inhibitor	Small molecule	Anticoagulant	Dipyridamole	Antilipophilic; channel coating	[97, 156, 157]

Zinc and zinc metalloenzymes are further alternative targets for drug development against SARS-CoV-2 entry. Facilitating zinc entry is a proposed mechanism for chloroquine and hydroxychloroquine, which are controversial antimalarial drugs repurposed for COVID-19 treatment as part of the SOLIDARITY trial. Acting as an ionophore, they increase intracellular lysosomal zinc concentrations, which has been shown to inhibit viral RNA-dependent RNA polymerase [63, 64]. They also raise the pH of lysosomes, thereby preventing their acidification and fusion with endosomes [65]. These drugs also impair the terminal glycosylation of ACE2, affecting its ligand binding ability [65, 66]. Despite this promising cell biology evidence, antimalarials did not show clinical benefit during selective clinical trials [67], prompting the need to improve the sophistication of current preclinical testing methods. Other antiviral drugs target viral replication after cell entry, such as remdesivir that is an RNA polymerase inhibitor developed for Ebola, or ritonavir and lopinavir, which are HIV protease inhibitors [65, 68]. Cathepsin-mediated cleavage of viral protein for processing is also a potential drug target being investigated, with certain compounds shown to have antiviral effects [65, 69]. Some of the aforementioned drugs can also benefit from nano-drug delivery modifications to improve their bioavailability and targeting [70, 71]. Combinations of drugs may also have synergistic effects potentially critical for overcoming SARS-CoV-2 infection [72, 73]. When designing lung MPS for drug combinations, it will be important to consider each component drug with regard to the design aspects discussed in “Physiologically and Pathologically Relevant Lung Models for COVID-19” and “Current challenges and future perspectives”.

### Inflammation

The inflammatory component of COVID-19 is a complex response to infection by SARS-CoV-2 in vascular connective tissue. A sequence of cell migration into the extracellular matrix occurs due to viral infection—this sequence begins with neutrophils followed by lymphocytes and macrophages [74, 75]. Inflammation also continues to play a role in the pulmonary edema occurring during the late acute/intermediate phase (Fig. 2), when fluid and plasma proteins followed by immune cells are exuded into the extracellular matrix. Anti-inflammatory treatments are frequently used to prevent the harmful effects of inflammation, which include fibrosis. Inflammatory cytokine release upon stimulation was shown to be higher in an airway-on-a-chip model compared to monoculture devices harboring the same type of cells, thus providing evidence of proinflammatory crosstalk in an MPS [76, 77]. The cytokine storm or cytokine release syndrome (CRS) is a robust cytokine release response, resulting in a hyperinflammatory state that can lead to life-threatening acute respiratory distress syndrome (ARDS) [78]. Eicosanoids, such as prostaglandins and leukotrienes generated from cell death debris, may be a trigger of the cytokine storms seen in COVID-19 [79]. Macrophage activation syndrome (MAS) and hemophagocytic lymphohistiocytosis (HLH) are autoimmune dysfunctions closely tied to the aberrant cytokine release in COVID-19 [80–82]. The tumor necrosis factor alpha (TNF-α) is a proinflammatory cytokine released by macrophages causing vascular leakage, edema and lung injury in COVID-19. As a result, the treatment of MAS with TNF-α inhibitors such as etanercept (a fusion protein between TGFR and IgG1 F_c_) is being explored [80, 83–85]. Blocking the cross-specificity of spike protein for Toll-like receptor 4 (TLR-4) has been proposed as a mechanism for the use of TLR-4 antagonists, such as eritoran, against TNF-α-mediated cytokine storm [86, 87]. The risk of opportunistic infections secondary to COVID has increased caution against immunomodulators and other biologics [80, 83, 88]. Sphingosine-1-phosphate (S1P) is a sphingolipid second messenger of the early innate immune response in the lung, whose suppression reduces TNF-α secretion and accompanying cytokine storm. The sphingosine-1-phosphate receptor (S1PR) antagonist fingolimod (Fig. 4a2, Table 1) is in phase II clinical trials for COVID-19 [78, 89]. Another important cytokine is IL-6, which binds to the IL-6 receptor on the cell surface and phosphorylates mediators in the Janus-associated kinase/signal transducer activator of transcription proteins (JAK/STAT) pathway, thus leading to the further upregulation of proinflammatory cytokines [89]. Tocilizumab and sarilumab (Table 1) are monoclonal antibody immunosuppressive drugs targeting the IL-6 receptor and antagonizing its ligand binding [80, 82, 89]. Baricitinib, ruxolinitinib (Fig. 4a1, Table 1) and tofacitinib are small-molecule immunosuppressants that inhibit the JAK/STAT pathway intracellularly and have been suggested for use against COVID-19-associated HLH [80, 81, 83, 89, 90]. Inflammation mediated by IL-6 is also thought to be closely linked to elevated ferritin levels found in non-survivors with severe COVID-19 [91–93]. IL-12 parallels IL-6 as a proinflammatory cytokine; thus, treatment with IL-12 inhibitors like ustekinumab has also been suggested [83, 85]. On the other hand, IL-10 presence indicates the beginning of the repair phase of inflammation and is associated with the non-inflammatory macrophage type 2 (M2) state, while its circulating levels are also elevated during a cytokine storm [84, 85]. In the RECOVERY trial, the corticosteroid dexamethasone (Table 1) was shown to reduce deaths in ICU patients and shorten hospital stays in non-ICU patients. Nanomedicine formulations of dexamethasone have been suggested for COVID-19, because its anti-edema and antifibrotic properties justify improved circulation time and targeting. In a clinical trial for multiple myeloma, PEGylated liposomal dexamethasone was well-tolerated [94]. In order to effectively model inflammation in lung MPS, the inclusion of cytokines, T-cells, neutrophils and other immune cells will likely capture a more complex phenomenon resembling the in vivo pathology. The length modeling of immune action in the lung is covered in “Modeling immunity in the lung”.

### Coagulation

The increased production of proinflammatory cytokines activating the coagulation reaction cascade leads to coagulopathy in COVID-19 patients. The coagulopathy seen in COVID-19 has the characteristics of sepsis-induced coagulation (SIC), as well as of severe disseminated intravascular coagulation (DIC) [95, 96]. The COVID-19 condition mildly prolongs prothrombin time (PT) and activated partial thromboplastin time (aPTT) and markedly elevates D-dimers. Activated coagulation indicators overlap but do not directly align with DIC and suggest a distinct hypercoagulable state [97], also termed COVID-19-associated coagulopathy (CAC) [92]. Integrating the microfluidic detection of hemostasis parameters [98] is a design consideration for an MPS model that includes coagulopathy. Venous thromboembolism (VTE) and pulmonary embolism (PE) are significant causes of mortality in COVID-19 patients and are thought to be caused by coagulopathy and further exacerbated by factors such as prolonged bed rest [96, 97, 99]. The prophylactic use of anticoagulants includes drugs such as tissue plasminogen activator (tPA), directly acting oral anticoagulants, dipyridamole, eculizumab and heparins like UFH, enoxaparin or dalteparin (Table 1) [95–97, 99, 100]. Heparins are also beneficial for their anti-inflammatory effects [93], with a poorly understood mechanism of action that may involve heparin binding and sequestration of cytokines. Thrombin activates PAR-1 augmenting inflammation, which decreases physiological anticoagulants such as antithrombin, thereby worsening coagulopathy [101]. In an alveolar MPS model of thrombosis where lipopolysaccharide (LPS) endotoxin was used to induce a prothrombotic cytokine cascade in the epithelium, the testing of PAR-1 inhibitor parmodulin-2 (PM-2, Table 1) demonstrated its cytoprotective and antithrombotic activity [77, 102].

### Pulmonary edema

At equilibrium, the active removal of liquid from the alveoli equals the inflow due to Starling forces, and the depth of liquid in the alveolar lumen is constant at approximately 0.1 µm [103]. Amiloride-sensitive Na^+^ absorption drives the removal of liquid from the alveolar lumen [104]. Anions (mainly Cl^–^) follow the actively absorbed Na^+^, resulting in a local osmotic gradient across the epithelium and water flowing along this gradient, which occurs primarily though alveolar type I cells that have the highest osmotic permeability of any cell type [105]. It is noteworthy that although human-induced pluripotent stem cell (iPSC)-derived alveolar epithelial cells express apical markers, such as human type I cell-associated protein of 56 kDa (HTI-56) and human type II cell-associated protein of 280 kDa (HTII-280), their aquaporin expression is varied [106, 107]. If there is outright damage to the epithelium or if its tight junctions become excessively leaky, however, the generated gradient is overwhelmed and flooding occurs. The loss of surfactant function also leads to alveolar flooding [108]. Lung-on-a-chip studies directed at pulmonary edema generated by IL-2 treatment for lung cancer demonstrated that the TRPV4 ion channel regulates the alveolar capillary barrier [109]. The TRPV4 inhibitor GSK2798745 (Table 1) is undergoing phase I clinical trials and has been suggested to be repurposed to treat pulmonary edema in COVID-19 [110].

### Fibrosis

Viral-induced pulmonary fibrosis contributes to both an immediate reduction in lung function and an increased risk of developing pulmonary fibrosis [111]. Epithelial cells within the lungs can proliferate following tissue damage to regenerate the native alveolar structure. However, inflammation or significant injury leads to proinflammatory signaling that activates fibroblasts, which aberrantly migrate and expand within the lung tissue to deposit stiff, collagen-rich ECM [112]. Epithelial cell apoptosis and the accumulation of fibrous scar tissue in conjunction inhibit proper lung function and in conjunction contribute to chronic symptoms. Fibrotic mechanisms should be incorporated into lung-on-a-chip systems to more faithfully mimic in vivo responses to viral-induced injury and better predict regenerative outcomes [113]. For therapeutic evaluation, lung-on-chip models typically utilize combinations of pirfenidone and/or nintedanib to remedy myofibroblast ECM production, which are intriguingly already under consideration as potential COVID-19 therapeutics [111].

A fibrotic phenotype is typically achieved by the addition of a lung fibroblast population and a fibrosis activation process (TGF-β supplementation) within type I collagen-based matrices and can be quantified in different ways depending on the system. In one approach, human small airway epithelial cells (SAECs) were combined with endothelial cells and normal lung fibroblasts within a multi-layer perfusable PDMS device with a distinct design that facilitates cell–cell interactions [114]. A fibrotic phenotype was induced via the addition of pulmonary fibrosis patient-derived fibroblasts and/or TGF-β and characterized via changes in smooth muscle actin (α-SMA), Tub4 and club cell uteroglobin (CC10) staining. Different custom approaches utilizing silk–collagen scaffolds were able to mimic the stiffening and alignment of fibroblasts within lung tissue observed in fibrotic disease progression; α-Al, ED-A fibronectin and periostin were used to identify the generation of myofibroblast populations [115]. In an alternative approach to study fibrotic stiffening, Asmani et al. developed a micropost system to hold epithelial–fibroblast microtissues. The relative movement of the posts in response to stretching could be used to determine tissue stiffness and compliance [116]. Following TGF-β induction, a fivefold increase in tissue stiffness was observed, mediated by increases in α-SMA and pro-collagen. Using this platform, based on the timing of TGF-β induction, pirfenidone and nintedanib were evaluated as both a preventative and treatment option for fibrosis. According to the markers and mechanical assessments of their model, a specific dose of pirfenidone (5.3 mM) was remarkably found to best counteract the fibrotic tissue phenotype.

## Modeling immunity in the lung

The cooperative responses of innate and adaptive immune cells within the lungs provide antibacterial, antifungal and antiviral immunity and are a source of significant paracrine signaling. Local macrophages and lymphocytes make up 90% and 10% of effector immune cells, respectively, within the lung parenchymal tissue [117]. Alveolar macrophages nonspecifically ingest foreign particles and, if a pathogen is identified, are responsible for the transfer antigen presentation to lymphocytes in the lymph nodes (APCs). In the presence of an antigen, chemokines produced by macrophages invoke adaptive immunity by recruiting lymphocytes to the specific locations of inflammation [86]. Tissue-resident cytotoxic T cells play a key role in antiviral immunity and have direct relevance to studies on SARS-CoV-2 infection therapy. The adaptive response type is defined by the immunomodulatory cytokine secretion profile. The induction of a primarily T helper 1 (IL-2, IFN-γ, TNF-α and GM-CSF) or T helper 2 (IL-4, IL-5, IL-9, IL-10 and IL-13) response affects the subsequently activated cell population, pathogen clearance mechanism and type of tissue damage [118]. The secretion of immunomodulatory factors from lung epithelial cells in response to stimulatory antigens also plays a critical role in defining the extent of immune response [119].

Recent efforts have aimed at recreating specific immune–epithelial cell interactions within lung-on-a-chip systems to better model intrinsic behaviors. In one specific study, primary bronchial or small airway epithelial cells were seeded on collagen-coated Transwell membranes and cultured under perfusion with an air–liquid interface to create matured upper or lower respiratory tract models, respectively [120]. Antigen-presenting cells, namely dendritic cells or macrophages, and fungal pathogens were seeded onto the membrane to evaluate the response of these immune cells within the epithelial network. The presence of pathogens in this model rapidly increased epithelial RANTES and IL-8 expression (within 30 min), induced dendritic cell maturation and migration through the tissue, and led to macrophage phagocytosis. This model can be expanded for infections with other airborne pathogens and subsequent evaluations of immune responses [121, 122]. In a different approach, Benam et al. incorporated mature airway epithelial cells into an air–liquid interface and an endothelial layer that separates the differentiated epithelium from flowing medium within a microfluidic device [76]. In this model, neutrophils could be introduced via the media channel and the resulting adhesion to the endothelial membrane could be evaluated. The delivery of viral RNA mimics polycytidylic acid into the matured device increased levels of RANTES, IL-6 and IP-10, which were markedly higher when both epithelial and endothelial cell populations were present, which elucidates the important role of pulmonary vasculature in mimicking intrinsic immune responses more closely. The application of this system for chronic obstructive pulmonary disease (COPD) epithelial cells with IL-13-induced asthma, or PLA-induced respiratory infection models, was used to evaluate therapeutics. The primary goal of these treatments is to reduce the extent of immune system overactivation featured in COPD patients. The observed reduction in neutrophil adhesion, adhesion marker mRNA levels and inflammatory secretion was sufficient to demonstrate differences between treatment with budesonide (Fig. 4a3), which has been noted as clinically ineffective for COPD, and a novel bromodomain-containing protein 4 (BRD4) inhibitor [76]. This lung device was further expanded to model the induced inflammatory environment following viral infection in asthmatic patients. By increasing the pore size of the cell-seeded membrane to 3 µm, the transmigration of flowing neutrophils could be evaluated. Changes due to an IL-13-induced asthma phenotype and infection with human rhinovirus 16 were evaluated based on cellular remodeling, temporal measurements of cytokine secretion (independently measured from the epithelial layer and media efflux) and neutrophil behaviors. This platform was sufficient to capture the altered immune response of asthmatic patients and to evaluate the therapeutic efficacy of a treatment that reduces neutrophil migration [123].

Bronchus-associated lymphoid tissues are found within the bronchioles and act as the local sites of B and T cell accumulation, antigen presentation and activation and consequent differentiation [117]. This process is dependent on both myeloid and plasmacytoid dendritic cells within the airway epithelium, which engulf antigens and, upon activation, migrate to the lymph nodes for antigen presentation [118, 124, 125]. Certain studies have explored the use of plasmacytoid dendritic cell-secreted interferon to counteract the SARS-CoV and SARS-CoV-2 suppression of innate immunity [126, 127]. Interestingly, the interferon-induced enzyme hydroxylation of cholesterol was shown to block viral replication [128], whereas a different study regarded cholesterol as important for viral entry [129]. Artificial lymph node structures and functions have been engineered through independently developed platforms, typically via immune cell encapsulation within hydrogel matrices under flow [130]. Compartmentalized dendritic, B-, and T-cell populations [131], antigen presentation and lymphocyte activation [132], and vaccination-mediated antibody production responses from patient-derived cells [133] have been demonstrated in various models. The introduction of relevant immune cells and secondary lymphoid-like tissues within lung-on-a-chip models would provide more biologically relevant responses to antigens and therapeutic treatments, interactions with endogenous cell types, and enable studies of vaccine development via the in vitro generation of antigen-specific antibodies and memory lymphocytes.

## Physiologically and pathologically relevant lung models for COVID-19

### Design criteria

In order to best evaluate therapeutics against COVID-19 and its associated tissue trauma, the development of lung models must combine design criteria spanning relevance to (1) native tissue structure and function and (2) disease-specific or multi-disease pathology. Based on our review of relevant scientific literature, we believe that the modular platform of lung MPS is the most promising candidate, owing to the capacity of incorporating multiple cells types, biochemical and biophysical stimulation, and diverse characterization and output parameters in 3D microenvironments [12]. Based on previous reports, in order to induce the distinct multi-cell differentiation present in native lung tissue, suggested components include: lung airway epithelial cells, endothelial cells and collagen-based matrix in the presence or absence of an air–liquid interface, liquid flow and stretching stimuli. The use of PDMS or other materials to house the tissue with micron-size features has demonstrated consistent success for incorporating single-circuit or multi-circuit microfluidic devices. Ongoing work seeks to make these devices easily manufactured at commercial scales, higher throughput with in situ spatiotemporal analysis, and from patient-derived cell types.

As previously discussed, novel models that integrate SARS-CoV-2 viral entry, immune responses and disease-specific pathology will be the best option to evaluate therapeutics across early- to late-stage infections. The first step is to employ lung primary cells (ex. alveolar type II) or cell lines that balance physiological relevance with ACE2 and TMSPR2 expression. Functions of inflammation and immunity can be introduced via exogenous cytokines (TNF-α, IL-6, IL-12), macrophages, neutrophils and/or lymphocytes. A key design component, via either membrane pores or compartmentalization, is the capacity for immune cell migration and maturation to enact direct and paracrine effects on lung tissue cells. We earlier discussed the most prevalent complications associated with COVID-19, namely coagulation, pulmonary edema and fibrosis. A corresponding disease-specific phenotype can be introduced and modeled via biochemical stimulation (LPS, IL-2, or TGF-β, respectively), additional cell types, genetic modifications, or changes in material stiffness. The direct integration or circuit connection of multiple disease elements should allow for therapeutic intervention and quantifiable disease outcomes for treatment evaluation in a higher-throughput fashion.

The integration of these intricate systems within a single tissue model represents a formidable challenge. However, COVID-19, which is currently affecting a rather large portion of the population with relatively unexplored long-term side-effects, justifies these efforts. An ideal system that can accurately determine effective therapeutics for each disease stage is therefore expected to have a significant impact.

### Lung MPS in a multi-system MPS

While lung microphysiological systems play an important role in testing leads for COVID development, interfacing a lung MPS device with multi-organ MPS or body-on-a-chip is necessary for determining secondary and systemic effects [134]. Systemic adverse effects such as cardiotoxicity and hepatotoxicity are among the leading causes of post-market drug withdrawal [135]. Testing compounds in multi-organ MPS can catch adverse effects missed by lung-only or independent MPS setups. Lung MPS can be incorporated into MPSs that model the liver, heart, pancreas, gut, kidney, endothelium and brain [136–138]. This is particularly important for testing COVID-19 therapeutics because of the exacerbation of disease severity by cardiometabolic comorbidities. IL-6-mediated inflammation plays a role in both COVID-19-derived cytokine storm, and cardiometabolic pathologies in the heart, kidney, pancreas and systemic vasculature [87]. The allometric scaling of additive components must be considered when generating a multi-organ MPS [137, 139]. In the combination of organ and endothelium representing total body vasculature, scaling based on the cell number gave better metabolic performance compared to scaling calculated by surface area [139]. Efforts should also be made to conform measures of drug absorption in each compartment with metrics such as mucus partition coefficient and mucosal epithelial permeability [140]. Furthermore, relating measures to physiological parameters such as TEER would allow interchangeability between cell types and facile the introduction of additional organ system compartments.

## Current challenges and future perspectives

The process of creating an in vitro model of COVID-19 infection presents unique challenges. First, the cellular components need careful selection. For instance, the SARS-CoV-2 infection induces cellular damage and an aberrant immune response leading to microenvironmental changes seen in the vascular compartment of the MPS, including elevated glucose, raised creatinine and markedly increased overall protein levels due to cell debris [141]. Although commercial media formulations including high glucose variants and elevated protein can be reproduced by the heat inactivation of serum, creatinine is often overlooked in vitro. There is evidence demonstrating that TLR-4 (Table 1) can be downregulated in RAW 264.7 macrophage cells due to elevated creatinine levels [142]. Additional considerations include whether the chosen cell types express the molecules in the target pathway in sufficient quantities for detection, or if the cells need to be further manipulated [22–24]. In addition to ensuring that cells chosen for the MPS endogenously express all components of the target pathways, they must be able to proliferate and achieve the desired phenotype and tissue organization on the substrate within the MPS microenvironment.

On the other hand, the biophysical properties of MPS should be tailored to mimic the in vivo tissue environment. For instance, there are significant differences in the elasticity of the components of lung MPS models, including the PDMS sidewalls, the material supporting the basement membrane of the alveolar epithelial and endothelial layers, as well as the cells themselves and their deposited ECM. Therefore, it should be considered that the elasticity changes with the disease state in some of these components. Lung elasticity is determined by the elastin content, patterning and the level of collagen isotypes [143–148]. The restructuring and remodeling of ECM is dependent on matrix metalloproteinases (MMP) regulated by tissue inhibitors of metalloproteinases (TIMP) [144, 149, 150] and involves the collagens to a much greater extent than elastin [145]. The ability to vary the elasticity of the substrate is especially important in a COVID model, since ARDS induces specific patterns of lung remodeling initiated by macrophages and continued by myofibroblasts [144], and ECM stiffening is a characteristic of senescence caused by interstitial fibrosis, alveolar septal cell loss and septal thinning [115, 116, 143, 151]. The ECM structure also influences the regulation of inflammation, since ECM pore size can affect the M1/M2 differentiation of macrophages [152]. One way of varying lung ECM in MPS is to use dynamically tunable biomaterials that allow for a noninvasive and temporal control of mechanical properties [153–155]. Alternatively, high-throughput platforms enabling the side-by-side comparison of various conditions (elasticity, micro-/macro-structure and chemical components) can be developed to simulate the changes and functional differences under physiological and pathological conditions.

The surface properties of MPS materials also need to be considered. Polydimethylsiloxane has many desirable properties, such as transparency, gas permeability, flexibility, ease of device manufacture, as well as biodegradability [156]. However, issues of unpolymerized oligomer leaching, absorption of small molecules, adsorption of proteins to hydrophobic surfaces and biofouling may limit its use in MPS models for screening drug candidates. A significant portion of unpolymerized oligomers can be removed by Soxhlet extraction or thermal aging [156, 157]. The surface binding of small molecules (200–400 kDa) is well-characterized and primarily determined by the molecule’s octanol–water partition coefficient (LogP > 4). Sol–gel methods and parylene coatings can reduce lipophilic molecule absorption [156, 157]. The Langmuir–Freundlich isotherm [158, 159] is a useful quantitative technique for predicting channel adsorption across a range of concentrations that could be encountered while testing a particular lead. The Langmuir–Freundlich isotherm is described by the following formula:

$${q}_{\mathrm{e}}=\frac{{Q}_{\mathrm{m}}{\left({K}_{\mathrm{a}}{C}_{\mathrm{e}}\right)}^{n}}{1+{\left({K}_{\mathrm{a}}{C}_{\mathrm{e}}\right)}^{n}},$$

where *q*_e_ (mg/g) represents the amount of compound (as solute) adsorbed, *Q*_m_ (mg sorbate/ g sorbent) represents the maximum adsorption capacity, *K*_a_ (L/mg) represents the affinity constant for adsorption, *C*_e_ represents the concentration at equilibrium (mg/L) and *n* represents the index of heterogeneity of the surface. The relation is semiempirical, allowing the aggregation of complex effects in a functional MPS. Alternative materials that exhibit low drug binding, such as polysulfones, polycarbonates and thermoplastics, can also be used in place of PDMS [160, 161]. Biologics such as antibodies and various other proteins can adsorb onto untreated PDMS surfaces, which reduces the concentration of analyte [162–164]. Treatment with oxygen plasma or other high energy methods is frequently used to increase hydrophilicity; however, the process is transient and the surface reverts in 15 min [157, 161]. Longer-term surface passivation of PDMS can be achieved by physical adsorption of amphiphilic molecules, covalent modification of self-assembled monolayers or grafting hydrophilic polymers such as polyethylene glycol (PEG), poly(N-isopropylacrylamide) (PNIPAAm) or 2-hydroxyethylmethacrylate (HEMA) [161]. Multiple coatings can also be applied; a PDMS surface coated with polyelectrolyte multilayer (PEM) and subsequent covalent attachment of PEG resulted in resistance to protein adsorption from rat serum for five months [157]. Alternative materials can also reduce biofouling. Promising elastomers such as PTCB-isobornyl exhibit excellent resistance to biofouling through their hydrophilicity and mechanical properties comparable to PDMS [162].

The conceptual basis of microfluidic flow, i.e., when small volumes are driven through bifurcating micron-scale channels, is readily applied to high-throughput screening. In early cancer modeling chip systems, 100 wells of HeLa cells arranged in a 10 × 10 array were subjected to a microfluidically generated concentration gradient of analyte for dosage–response analysis [163]. Recently, up to 4000 wells of cells have been fabricated on a single device [164]. However, many such systems are monocultures that lack the complex tissue architecture that is important for an effective in vitro lung model. Human iPSC-derived lung organoids were lately used to screen drugs that blocked SARS-CoV-2 viral entry in ACE-2-expressing cells. The study showed that imatinib, mycophenolic acid and quinacrine dihydrochloride inhibited viral infection [165]. As discussed earlier, an MPS model of the upper airway was multiplexed into 96 wells for screening anti-fibrotic agents. This MPS device recreated an air–liquid interface, giving component cells a physiologically accurate microenvironment [115]. While pumpless and pump-driven microfluidics in MPS devices inherently simplify the bottleneck steps of liquid handling, integrating MPS with recent advances in automated liquid handling and continuous flow can further enhance throughput [166–168], as well as facilitate biocontainment for studying viral disease [138].

The role of artificial intelligence (AI) in accelerating drug development and repurposing is an intriguing field that has attracted much interest. In other COVID-19-related areas, artificial intelligence and machine learning have proven useful for applications such as molecular design, data mining and image analysis [169]. Convolutional neural network (CNN) powered image analysis could be applied to the detection of characteristic viral cytopathology in an MPS without fixation and staining [170]. Integrating such enabling technology would likely accelerate the throughput of lung MPS experiments. The use of AI for synergistic effects of drug combinations is an actively explored topic [74, 171]. While methods utilizing graphics processing units (GPU) have accelerated molecular docking studies [9], their accuracy is still dependent on the scoring function used. The use of AI algorithms, such as random forest and neural networks, improves the accuracy of scoring functions and enables progress toward the rapid and accurate detection of protein–ligand pairs [166, 172]. Artificial intelligence has been applied to determine the affinity of ligands to target SARS-CoV-2 proteins, though it can also be used to predict the toxicity of a given drug via training on publicly available toxicity datasets (TOXNET or ToxCast) and empirically or structurally calculated molecular descriptors [173]. Adversarial autoencoder (AAE) algorithms have been used to design novel inhibitors based on existing templates and desired gene expression profiles [174]. Although quite powerful, AI faces many barriers to implementation in drug development that AI proponents are trying to overcome [175]. A solid strategy for data-driven drug development includes integration with in vitro preclinical validation [176]. A lightweight in vitro AI validation method in the form of lung MPS devices will certainly help accelerate drug development against the current pandemic.

## Conclusions

The application of MPS to understand lung physiology and disease states is expanding at a rapid pace. Their role alongside other co-culture systems, such as organoids and Transwell systems, in drug discovery that more closely follows in vivo physiology and pathology, should become the next rising trend. The efficient adaptation of these in vitro models for the development and characterization of COVID-19 treatments will likely benefit patients on an unparalleled scale and lay the groundwork toward preparedness for the future, where the increasing global connectedness could lead to pandemics of similar or even greater magnitude than that of the current coronavirus outbreak.
